# Generic and vision related quality of life associated with different types of cataract surgeries and different types of intraocular lens implantation

**DOI:** 10.1371/journal.pone.0240036

**Published:** 2020-10-02

**Authors:** Shalu Jain, Akshay Chauhan, Kavitha Rajshekar, Praveen Vashist, Promila Gupta, Umang Mathur, Noopur Gupta, Vivek Gupta, Parul Dutta, Vijay Kumar Gauba

**Affiliations:** 1 Health Technology Assessment in India, Department of Health Research, Ministry of Health and Family Welfare, New Delhi, India; 2 Community Ophthalmology, Dr. R. P. Centre for Ophthalmic Sciences, All India Institute of Medical Sciences, Ansari Nagar, New Delhi, India; 3 National Programme for Control of Blindness & Visual Impairment, Ministry of Health & Family Welfare, Nirman Bhawan, New Delhi, India; 4 Dr. Shroff’s Charity Eye Hospital, Daryaganj, New Delhi, India; National Eye Institute, UNITED STATES

## Abstract

**Objectives:**

To assess the effects of different types of cataract surgeries and intraocular lenses on generic as well as vision related quality of life of cataract patients, using EQ-5D and IND-VFQ 33 instruments respectively.

**Methods:**

An observational, longitudinal study of patients undergoing cataract surgery was carried out at three ophthalmology centres. Patients were prospectively admitted for surgery for age-related cataract. Generic quality of life was assessed by using Euroqol’s EQ5D-5L questionnaire and vision related quality of life was assessed by the IND-VFQ-33 questionnaire. Data pertaining to vision function and quality of life were collected pre surgery and 4 weeks after the surgery.

**Results:**

Out of total patients (n = 814) recruited for the study, 517 patients were interviewed for both pre-surgery and post-surgery for EQ5D and 519 patients were interviewed for both pre-surgery and post-surgery for IND VFQ 33 tool. The combined data from all three centres showed that Quality Adjusted Life Year (QALY) gains observed in patients undergoing phacoemulsification with foldable lens implantation (2.25 QALY) were significantly higher (0.57 QALY) as compared to Small Incision Cataract Surgery (SICS) with PMMA lens implantation (1.68 QALY). Highest improvement however, in all three subscales of IND-VFQ-33 tool were clearly observed for SICS with PMMA lens implantation.

**Conclusions:**

The study has elicited the Health related and vision related Quality of Life scores for cataract surgeries and subsequent lens implantation. This study also offers Health State Utility Values along with visual outcomes for different surgical procedures, lenses and for the combination of surgery with lens implantation for cataract procedures providing a useful resource for future economic evaluation studies.

## Introduction

Health Technology Assessment in India (HTAIn) has been institutionalised since 2016–17 under Department of Health Research (DHR), Ministry of Health and Family Welfare (MoHFW) by the Government of India to facilitate the process of transparent and evidence-based decision making for better healthcare delivery [1–5]. Health Technology Assessment (HTA) is an internationally accepted tool to inform decision making for better management of existing resources for Universal Health Coverage (UHC) [6–8].

Rashtriya Swasthya Bima Yojana (RSBY), launched in 2008, was one of the most significant centrally funded efforts towards providing UHC in India [9]. However, RSBY scheme was later subsumed by the Ayushman Bharat -Pradhan Mantri Jan Aarogya Yojna (AB-PMJAY) [10]. AB-PMJAY was launched in early 2018, by the Government of India to provide comprehensive cover for primary, secondary, and tertiary care amenable conditions [11, 12]. Cataract procedures were the most commonly claimed packages under RSBY with lot of ambiguities in the packages provided under the scheme. It was therefore an urgent requirement for the policymakers to fix cataract packages to be covered under the new AB-PMJAY scheme.

Therefore, the first HTA study undertaken by the HTAIn secretariat was “health technology assessment on intraocular lenses for treatment of age-related cataracts”. Aim of this HTA study was to assess the cost-effectiveness of benefit packages for treatment of age-related cataract using various types of Intra-Ocular Lenses (IOLs) over a lifetime horizon for the Indian population using a health sector as well as societal perspective. This HTA study involved five individual literature reviews to gather the existing evidences on different aspects, including clinical effectiveness, cost effectiveness, Health Related Quality of Life (HRQoL), costing, and equity pertaining to different types of cataract surgeries being performed and IOLs being implanted in India [5, 13].

The surgical options available for cataract surgery in India are Intra- Capsular Cataract Extraction (ICCE), Extra- Capsular Cataract Extraction (ECCE), Small-Incision Cataract Surgery (SICS) and phacoemulsification (Phaco) [14]. Cataract surgery by ICCE is declining rapidly [15]. Large incision ECCE is still being performed for certain cases of cataract where other techniques are either not feasible or not available [13]. However, the most commonly performed surgeries in India are Phaco and SICS. Currently 90% of all the cataract surgeries are performed with Intraocular lenses [16]. IOLs can be made up of a range of different materials [17]. Polymethylmethacrylate (PMMA) IOLs are inflexible, require a larger incision for implantation (5–7 mm requiring sutures) compared with newer foldable silicone (hydrophobic) and acrylic (hydrophobic or hydrophilic) lenses (2.2–3.5 mm and not requiring sutures) [18].

In clinical decision-making, interventions are being primarily assessed based on efficacy and safety. However, in cost effectiveness analysis where effects are considered in the form of Quality adjusted Life Years (QALY), it is also important to monitor the impact that treatments have on health state utility values (HSUVs) i.e., health-related quality of life (HRQoL) using validated instruments [9, 10]. HSUVs can be obtained from various measures e.g. condition-specific preference-based measures (CSPBMs) or generic preference-based measures (GPBMs) [19–22]. Generic measure can be used in a wide range of health conditions and treatments and allows comparison across different conditions whereas condition specific measures are supposed to be more responsive to the particular condition [19–22].

A systematic review conducted to collect HSUVs under the HTA study mentioned above, suggested that there were not enough studies comparing HRQoL between different type of cataract surgeries and lenses, especially in Indian context [5]. Moreover, the studies available were highly heterogeneous in terms of study design, population, instruments used for measuring the health states, value sets used for assigning utility weights, and reporting results. Therefore, a primary study was conducted to assess the impact on generic as well as vision related quality of life, different types of cataract surgeries (ECCE, SICS, Phacoemulsification) and lenses (rigid and foldable lenses) having on age related cataract patients.

The objective of this study was to assess how different types of cataract surgeries and lenses affects the generic as well as vision related quality of life using EQ-5D and IND-VFQ 33 instruments respectively. The aim was also to generate HSUVs to be incorporated in the HTA study on cataract procedures.

## Subjects and methods

### Study centres

This longitudinal, observational study was conducted at three ophthalmology centres. Dr. Rajendra Prasad Centre for Ophthalmic Sciences, All India Institute of Medical Sciences, New Delhi (AIIMS-Delhi) is an apex tertiary care institute of India which caters to patients coming from all over the country, provides facility for phacoemulsification surgery to all patients irrespective of their paying capacity. Hence, this centre was selected to ensure we get a mixed population sample representing different regions of the country. Another centre selected for the study was Dr. Shroff’s Charity Eye Hospital, New Delhi (Shroff Delhi). Shroff, Delhi is a non-government tertiary care organization that works on a cross-subsidy model, catering to all socioeconomic segments of society and provides both SICS and Phaco surgeries. Third centre selected was another branch of Dr. Shroff’s Charity Eye Hospital, located in Vrindavan (Shroff Vrindavan), a small town in Uttar Pradesh and provides mostly SICS for treatment of cataract. The centre was selected to ensure the patient sample also represent rural population of India. These three centres catered to a heterogeneous population pool and had exclusive cataract services with all the required facilities, within the scope of our study, for the patients.

### Subjects

Patients admitted for age-related cataract surgery were prospectively recruited in the study after getting approvals from AIIMS Institute Ethics Committee and Dr. Shroff’s Charity Eye Hospital ethics committee. Written informed consents were obtained from each patient to participate in the study. These patients underwent either ECCE or SICS or Phacoemulsification and had either rigid (PMMA) or foldable lens implanted. Based on this, we had following eleven groups or categories into which the patients were divided (Table 1).

**Table 1:** Categorization of recruited patients based upon the type of surgery and lens implantation they had undergone.

Category	Description
**ECCE & Foldable**	Patients undergoing ECCE with foldable lens implanted
**ECCE & PMMA**	Patients undergoing ECCE with rigid (PMMA) lens implanted
**Phaco & Foldable**	Patients undergoing phacoemulsification with foldable lens implanted
**Phaco & PMMA**	Patients undergoing phacoemulsification with rigid (PMMA) lens implanted
**SICS & Foldable**	Patients undergoing SICS with foldable lens implanted
**SICS & PMMA**	Patients undergoing SICS with rigid (PMMA) lens implanted
**ECCE**	All Patients undergoing ECCE irrespective of which lens was implanted
**Phaco**	All Patients undergoing phacoemulsification irrespective of which lens was implanted
**SICS**	All Patients undergoing SICS irrespective of which lens was implanted
**Foldable**	All Patients implanted with foldable lens irrespective of the type of surgery they underwent
**PMMA**	All Patients implanted with rigid (PMMA) lens irrespective of the type of surgery they underwent

### Sample size

Considering the mean utility score of cataract patients in pre-operative and post-operative phase as 0.782 (SD 0.15) and 0.832 (SD 0.129) respectively from a study published from low income settings [23], the anticipated difference in the utility score from the known population as 10%, type-I error as 0.05, power of the study as 80%, and non- response rate of 20%, a sample size of 461 was estimated to be appropriate. Considering a follow up rate of 60% in Indian settings, this number was increased to 768 to account for 40% loss to follow-up cases. The aim was thus to recruit more than 768 cataract patients across the three facilities within the study duration.

### Data collection

All data were collected on standardised proforma. Clinical data (on the process of care provided and related clinical outcomes) were collected by the ophthalmologists concurrently with routine preoperative assessments and at dedicated postoperative follow up 4 weeks after the surgery. As the study aims to assess QoL gained by cataract surgery, data pertaining to vision function and quality of life were obtained from a standardised administered interview both pre and postoperatively wherein, both the QoL instruments used in this study (EQ5D and IND VFQ33) were executed either on the day of the surgery or one day before surgery depending upon the patient admission and stay in the hospital and both the instruments were again administered during the post-surgical follow-up, that is 4 weeks after the surgery.

### Generic quality of life

The instrument used for measuring generic QoL was Euroqol’s EQ5D-5L questionnaire. A prior approval has been sought from Euroqol Research Foundation to use the EQ5D-5L questionnaire. The questionnaire used (in Hindi and English languages) were provided by Euroqol Research Foundation upon a request made by authors. EQ-5D consists of a descriptive system and the EQ visual analogue scale (VAS). The descriptive system comprises five dimensions: mobility, self-care, usual activities, pain/discomfort and anxiety/depression [24]. The EQ VAS records the patient’s self-rated health on a vertical visual analogue scale ranging from 0 to 100, where 0 represents worst and 100 represent best health states [24].

Quality adjusted life years (QALYs) were estimated by using age adjusted life expectancy rates for India from Sample Registration Survey (SRS) life tables for 2012–16 [25]. Due to non-availability of Indian value sets at the time of this study, Indonesian EQ5D-5L value sets were used to assign quality of life weights against each health state [26].

### Visual function scores

The vision related QoL was measured by using 33-item Indian Vision Function Questionnaire (IND-VFQ-33) [27, 28]. This scale has been developed from focus group discussions with Indian patients; has a concise format; is easy to administer; and has been validated using traditional validation techniques such as classical test theory (CTT), and modern psychometric methods such as Rasch analysis [27–29]. IND-VFQ-33 has three independent subscales for general functioning, psychosocial impact and visual symptoms therefore individual composite scores were generated for each of the three parts of the questionnaire during the analysis.

### Visual outcomes

Preoperative, operative and postoperative data was collected on visual acuity. Data on visual outcome was grouped using WHO’s classification. All patients were categorized as having good, poor or borderline visual acuity with no surgical complications as a good outcome (visual acuity ≥6/18), a borderline outcome (visual acuity 6/24–6/60) and a poor outcome (visual acuity ≤6/60) [30]. Pre surgery visual acuity was compared with uncorrected visual acuity (UCVA) 4 weeks after the surgery in the operated eye. UCVA 4 weeks after the surgery was correlated with the QALY gains.

### Refractive outcomes

To assess the refractive outcome data, we have compared follow up visual acuity (4 weeks after the surgery) in operated eyes (UCVA vs BCVA).

### Statistical analysis

Data was analyzed either by using Microsoft Excel Worksheet (Microsoft Office 365), or by Stata 15 software. The QoL and QALY results are given as the mean values. The significance of the difference in utility before and after cataract surgery was analyzed with a paired t test. A P value of less than 0.05 was considered statistically significant.

## Results

### 1. Subjects

A total of 814 patients admitted for age-related cataract surgery were prospectively recruited in the study from three different centres. The mean age of the patients undergoing surgery was 60.56 years (Table 2).

**Table 2:** Distribution of recruited patients at different centres with average age in years.

	Overall	AIIMS	Shroff Delhi	Shroff Vrindavan
	Number (%)	Number (%)	Number (%)	Number (%)
	(Average Age in years)	(Average Age in years)	(Average Age in years)	(Average Age in years)
Males	386 (47.4%)	171 (43.3%)	89 (42.6%)	126 (60%)
	60.58	60.04	60.55	61.65
Females	428 (52.6%)	224 (56.7%)	120 (57.4%)	84 (40%)
	60.25	60.13	61.04	62.2
Total	814	395	209	210
	60.58	60.04	60.55	61.65

### 2. Post-surgery follow up of patients

Out of total patients recruited for the study, overall post-surgical follow up rate was 63.8%, where 517 patients were interviewed for both pre and post-surgery for EQ5D and 519 patients were interviewed for both pre and post-surgery for IND VFQ 33 tool. Detailed distribution of patients according to the QoL instrument is given in Table 3.

**Table 3:** Distribution of patients-questionnaire wise.

**Category**	**EQ5D**	**VFQ**
	**Number (% of total patients recruited)**	
Patients followed up	517 (63.5%)	519 (63.8%)
**Category**	**Number (% of followed up patients)**	
**Type of Surgery**		
1. ECCE	31 (6%)	31 (6%)
2. Phaco	360 (70%)	361 (70%)
3. SICS	126 (24%)	127 (24%)
**Type of IOL**		
1. Foldable	333 (64%)	335 (65%)
2. PMMA	184 (36%)	184 (35%)
**Surgery & IOL**		
1. ECCE & Foldable	3 (<1%)	3 (<1%)
2. ECCE & PMMA	28 (5%)	28 (5%)
3. Phaco & Foldable	327 (63%)	330 (64%)
4. Phaco & PMMA	33 (6%)	31 (6%)
5. SICS & Foldable	3 (<1%)	2 (<1%)
6. SICS & PMMA	123 (24%)	125 (24%)

### 3. HRQoL results

HRQoL results are given below for both the tools (EQ-5D and IND-VFQ-33) separately. For each tool, results are presented for each centre individually and finally as combined pooled results. For EQ-5D results QALY gains are given as composite score of the five dimensions: mobility, self-care, usual activities, pain/discomfort and anxiety/depression of EQ-5D tool. For IND-VFQ-33, individual scores for each subscale of the tool (including general functioning, psychosocial impact and visual symptoms) are presented.

#### 3.1 AIIMS Delhi

AIIMS, Delhi performs ECCE and Phacoemulsification with either PMMA or foldable lens implantation for the cataract surgery with maximum procedures being Phaco with foldable lenses.

*EQ-5D-5L_ AIIMS Delhi Results*. Results shows that the gain in QALY in case of Phaco with foldable lens implantation (2.53 QALY) is slightly higher (0.03 QALY) as compared to the gain in QALY in case of ECCE with rigid lens implantation (2.5 QALY). (Table 4).

**Table 4:** EQ-5D results of AIIMS-Delhi.

N	Category	Mean									
		Age (Years)	Pre Utility	Post Utility	Change (QoL)	Pre QALY	Post QALY	Change (QALY)	Pre VAS	Post VAS	Change
**3**	**ECCE & Foldable**	59	0.76	0.69	-0.07	15.19	14.01	**-1.18**	**43.33**	**70**	**26.67**
**28**	**ECCE & PMMA**	60	0.77	0.9	0.14	14.98	17.49	**2.5**	**55.36**	**77.79**	**22.43**
**197**	**Phaco & Foldable**	60.03	0.78	0.9	0.11	15.06	17.59	**2.53**	**54.22**	**81.7**	**27.48**
**29**	**Phaco & PMMA**	60.28	0.83	0.84	0.01	15.64	15.86	**0.22**	**46.72**	**74.31**	**27.59**
**31**	**ECCE**	59.90	0.77	0.88	0.12	15.01	17.15	**2.15**	**54.19**	**77.03**	**22.84**
**226**	**Phaco**	60.06	0.79	0.89	0.1	15.13	17.37	**2.23**	**53.26**	**80.75**	**27.49**
**200**	**Foldable**	60.02	0.78	0.9	0.11	15.06	17.53	**2.48**	**54.06**	**81.53**	**27.47**
**57**	**PMMA**	60.14	0.80	0.87	0.07	15.32	16.66	**1.34**	**50.96**	**76.02**	**25.05**
**257**	**Overall**	60.04	0.79	0.89	0.1	15.12	17.34	**2.22**	**53.37**	**80.3**	**26.93**

*IND-VFQ-33_ AIIMS Delhi Results*. Positive change in visual symptoms and in psychosocial impact was found to be highest in case of Phaco with foldable lens implantation as compared to the other categories. (Table 5a).

**Table 5:** Table representing change in the IND-VFQ-33 scores pre and post-surgery for AIIMS-New Delhi (a), Shroff Delhi (b), Shroff Vrindavan (c), Shroff Combined (d), Combined All (e).

N	Category	Mean Pre surgery			Mean Post surgery			Post-Surgery Change		
		General	Psychosocial	Vision	General	Psychosocial	Vision	Change General	Change Psycho.	Change Vis.
**Table 5a AIIMS-New Delhi**										
3	ECCE & Foldable	78.17	90.00	67.86	93.65	98.33	76.19	15.48	8.33	8.33
28	ECCE & PMMA	88.44	92.14	63.90	95.14	99.29	85.33	6.70	7.14	21.43
197	Phaco & Foldable	88.45	89.95	56.82	97.69	99.06	89.54	9.23	9.11	32.72
29	Phaco & PMMA	89.24	90.86	53.69	92.90	97.24	80.67	3.65	6.38	26.97
31	ECCE	87.44	91.94	64.29	94.99	99.19	84.45	7.55	7.26	20.16
226	Phaco	88.55	90.07	56.42	97.07	98.82	88.40	8.52	8.76	31.98
200	Foldable	88.30	89.95	56.98	97.63	99.05	89.34	9.33	9.10	32.36
57	PMMA	88.85	91.49	58.71	94.00	98.25	82.96	5.15	6.75	24.25
257	Overall	88.42	90.29	57.37	96.82	98.87	87.92	8.40	8.58	30.55
**Table 5b Shroff-Delhi**										
111	Phaco & Foldable	72.05	69.77	41.36	93.11	95.54	89.19	21.06	25.77	47.83
17	SICS & PMMA	52.41	57.94	28.36	83.75	88.24	89.27	31.35	30.29	60.91
111	Phaco	72.05	69.77	41.36	93.11	95.54	89.19	21.06	25.77	47.83
17	SICS	52.41	57.94	28.36	83.75	88.24	89.27	31.35	30.29	60.91
111	Foldable	72.05	69.77	41.36	93.11	95.54	89.19	21.06	25.77	47.83
17	PMMA	52.41	57.94	28.36	83.75	88.24	89.27	31.35	30.29	60.91
128	Overall	69.44	68.19	39.63	91.87	94.57	89.19	22.43	26.38	49.57
**Table 5c Shroff-Vrindavan**										
22	Phaco & Foldable	55.14	57.73	62.66	97.40	96.14	95.13	42.26	38.41	32.47
2	Phaco & PMMA	37.50	55.00	60.71	50.00	70.00	67.86	12.50	15.00	7.14
2	SICS & Foldable	59.52	85.00	69.64	100.00	100.00	100.00	40.48	15.00	30.36
108	SICS & PMMA	40.85	40.57	55.86	97.64	98.37	94.54	56.80	57.80	38.68
24	Phaco	53.67	57.50	62.50	93.45	93.96	92.86	39.78	36.46	30.36
110	SICS	41.18	41.38	56.11	97.68	98.40	94.64	56.50	57.02	38.53
24	Foldable	55.51	60.00	63.24	97.62	96.46	95.54	42.11	36.46	32.29
110	PMMA	40.78	40.84	55.95	96.77	97.86	94.05	55.99	57.02	38.11
134	Overall	43.42	44.27	57.25	96.93	97.60	94.32	53.50	53.33	37.06
**Table 5d Shroff-Combined (Shroff Delhi+Shroff Vrindavan)**										
130	Phaco & Foldable	69.25	67.76	44.91	93.82	95.64	90.17	24.58	27.88	45.27
4	Phaco & PMMA	37.50	55.00	60.71	50.00	70.00	67.86	12.50	15.00	7.14
2	SICS & Foldable	59.52	85.00	69.64	100.00	100.00	100.00	40.48	15.00	30.36
123	SICS & PMMA	42.41	42.94	52.12	95.75	96.99	93.82	53.34	54.05	41.70
134	Phaco	68.78	67.57	45.14	93.17	95.26	89.84	24.40	27.69	44.70
125	SICS	42.68	43.60	52.40	95.82	97.04	93.92	53.13	53.43	41.52
132	Foldable	69.10	68.02	45.27	93.92	95.70	90.32	24.81	27.68	45.04
127	PMMA	42.34	43.13	52.25	95.03	96.56	93.41	52.69	53.43	41.16
259	Overall	56.12	55.94	48.67	94.46	96.12	91.82	38.34	40.18	43.15
**Table 5e Combined (AIIMS Delhi+Shroff Delhi+Shroff Vrindavan)**										
3	ECCE & Foldable	78.17	90.00	67.86	93.65	98.33	76.19	15.48 (<0.01)	8.33 (0.19)	8.33 (<0.05)
28	ECCE & PMMA	88.44	92.14	63.90	95.14	99.29	85.33	6.7 (<0.01)	7.14 (<0.01)	21.43 (<0.01)
330	Phaco & Foldable	80.73	81.04	52.03	96.12	97.67	89.80	15.4 (<0.01)	16.64 (<0.01)	37.76 (<0.01)
31	Phaco & PMMA	85.91	88.55	54.15	90.13	95.48	79.84	4.22 (<0.01)	6.94 (<0.05)	25.69 (<0.01)
2	SICS & Foldable	59.52	85.00	69.64	100.00	100.00	100.00	40.48 (<0.01)	15 (0.07)	30.36 (<0.01)
125	SICS & PMMA	42.41	42.94	52.12	95.75	96.99	93.82	53.34 (<0.01)	54.05 (<0.01)	41.7 (<0.01)
31	ECCE	87.44	91.94	64.29	94.99	99.19	84.45	7.55 (<0.01)	7.26 (<0.01)	20.16 (<0.01)
361	Phaco	81.17	81.69	52.22	95.61	97.49	88.94	14.44 (<0.01)	15.8 (<0.01)	36.72 (<0.01)
127	SICS	42.68	43.60	52.40	95.82	97.04	93.92	53.13 (<0.01)	53.43 (<0.01)	41.52 (<0.01)
335	Foldable	80.58	81.14	52.28	96.13	97.69	89.73	15.55 (<0.01)	16.55 (<0.01)	37.45 (<0.01)
184	PMMA	56.76	58.13	54.26	94.71	97.09	90.17	37.95 (<0.01)	38.96 (<0.01)	35.91 (<0.01)
519	Overall	72.13	72.98	52.98	95.62	97.48	89.89	23.49 (<0.01)	24.5 (<0.01)	36.91 (<0.01)

#### 3.2 Shroff Delhi

Shroff, Delhi performs SICS with PMMA lenses and Phacoemulsification with foldable lens implantation for the cataract surgery with maximum procedures being Phaco with foldable lenses.

*EQ-5D-5L Results_ Shroff Delhi*. The gain in QALY in case of SICS with rigid lens implantation (3.34 QALY) is significantly higher (1.42 QALY) as compared to the gain in QALY in case of Phaco with foldable lens implantation (1.92 QALY). (Table 6).

**Table 6:** EQ-5D results of Shroff-Delhi.

N	Category	Mean									
		Age	Pre Utility	Post Utility	Change	Pre QALY	Post QALY	Change (QALY)	Pre VAS	Post VAS	Change
**108**	**Phaco & Foldable**	60.06	0.88	0.98	0.11	17.46	19.39	**1.92**	**69.35**	**89.47**	**20.12**
**17**	**SICS & PMMA**	64.71	0.84	0.99	0.14	13.33	16.67	**3.34**	**68.65**	**93.82**	**25.18**
**108**	**Phaco**	60.06	0.88	0.98	0.11	17.46	19.39	**1.92**	**69.35**	**89.47**	**20.12**
**18**	**SICS**	63.89	0.85	0.99	0.14	14.08	17.24	**3.16**	**70.39**	**94.17**	**23.78**
**109**	**Foldable**	59.97	0.88	0.98	0.11	17.55	19.46	**1.91**	**69.63**	**89.57**	**19.94**
**17**	**PMMA**	64.71	0.84	0.99	0.14	13.33	16.67	**3.34**	**68.65**	**93.82**	**25.18**
**126**	**Overall**	60.61	0.87	0.98	0.11	16.98	19.08	**2.10**	**69.5**	**90.14**	**20.64**

*IND VFQ 33 Results_ Shroff Delhi*. Scores of all three subscales including general functioning, psychosocial impact and visual symptoms were found to be higher in case of SICS with rigid lens implantation as compared to the Phaco with foldable lens implantation. (Table 5b).

#### 3.3 Shroff Vrindavan

As mentioned in Table 1, Shroff Vrindavan, performs SICS and Phacoemulsification with either PMMA or foldable lens implantation for the cataract surgery with maximum procedures being SICS with PMMA lenses.

*EQ-5D-5L Results_ Shroff Vrindavan*. The gain in QALY in case of SICS with PMMA lens implantation (1.42 QALY) is slightly higher (0.04 QALY) as compared to the gain in QALY in case of Phaco with foldable lens implantation (1.38 QALY). (Table 7).

**Table 7:** EQ-5D results of Shroff-Vrindavan.

N	Category	Mean									
		Age	Pre Utility	Post Utility	Change	Pre QALY	Post QALY	Change (QALY)	Pre VAS	Post VAS	Change
**22**	**Phaco & Foldable**	60.05	0.91	0.98	0.07	17.34	18.72	**1.38**	**65.32**	**95.68**	**30.36**
**4**	**Phaco & PMMA**	67.75	0.95	0.97	0.02	12.79	13.11	**0.32**	**75.00**	**90.00**	**15.00**
**2**	**SICS & Foldable**	58.50	0.94	0.94	0.00	19.78	19.78	**0.00**	**52.50**	**100.00**	**47.50**
**106**	**SICS & PMMA**	61.64	0.90	0.98	0.08	15.98	17.40	**1.42**	**55.20**	**92.59**	**37.40**
**26**	**Phaco**	61.23	0.91	0.98	0.06	16.64	17.86	**1.22**	**66.81**	**94.81**	**28.00**
**108**	**SICS**	61.58	0.90	0.98	0.08	16.05	17.44	**1.39**	**55.15**	**92.73**	**37.58**
**24**	**Foldable**	59.92	0.91	0.98	0.07	17.54	18.81	**1.27**	**64.25**	**96.04**	**31.79**
**110**	**PMMA**	61.86	0.90	0.98	0.08	15.87	17.24	**1.38**	**55.92**	**92.50**	**36.58**
**134**	**Overall**	61.51	0.90	0.98	0.08	16.17	17.53	**1.36**	**57.41**	**93.13**	**35.72**

*IND VFQ 33 Results_ Shroff Vrindavan*. Scores of all three subscales including general functioning, psychosocial impact and visual symptoms were found to be highest in case of SICS with rigid lens implantation as compared to other categories. (Table 5c).

#### 3.4 Shroff combined (Delhi + Vrindavan)

If we combine the data from both the Shroff Centres (Delhi and Vrindavan), we get a good number of procedures for Phaco with foldable lens and SICS with PMMA lens, that allows a more reliable comparison for the two interventions.

*EQ-5D-5L Results_ Shroff Combined*. The combined data from two of Shroff centres shows that the gain in QALY for Phaco with foldable lens implantation (1.83 QALY) is higher (0.15 QALY) as compared to the gain in QALY for SICS with PMMA lens (1.68 QALY). (Table 8).

**Table 8:** EQ-5D results of Shroff combined data (Shroff Delhi+ Shroff Vrindavan).

N	Category	Mean									
		Age	Pre Utility	Post Utility	Change	Pre QALY	Post QALY	Change (QALY)	Pre VAS	Post VAS	Change
**130**	**Phaco & Foldable**	60.06	0.88	0.98	0.10	17.44	19.27	1.83	**68.67**	**90.52**	**21.85**
**4**	**Phaco & PMMA**	67.75	0.95	0.97	0.02	12.79	13.11	0.32	**75.00**	**90.00**	**15.00**
**2**	**SICS & Foldable**	55.67	0.96	0.96	0.00	22.15	22.15	0.00	**68.33**	**100.00**	**31.67**
**123**	**SICS & PMMA**	62.07	0.89	0.98	0.09	15.62	17.30	1.68	**57.06**	**92.76**	**35.71**
**134**	**Phaco**	60.29	0.88	0.98	0.10	17.30	19.09	1.78	**68.86**	**90.51**	**21.65**
**125**	**SICS**	61.91	0.89	0.98	0.09	15.77	17.42	1.64	**57.33**	**92.94**	**35.61**
**132**	**Foldable**	59.96	0.88	0.98	0.10	17.55	19.34	1.79	**68.66**	**90.74**	**22.08**
**127**	**PMMA**	62.24	0.89	0.98	0.09	15.53	17.17	1.64	**57.62**	**92.68**	**35.06**
**259**	**Overall**	61.08	0.89	0.98	0.09	16.56	18.27	1.71	**63.27**	**91.68**	**28.42**

*IND VFQ 33 Results_ Shroff Combined (Delhi + Vrindavan)*. Post-surgery positive changes in general functioning and psychosocial impact were found to be highest in case of SICS with PMMA lens implantation, whereas post-surgery positive changes for visual symptoms were highest in case of phaco with foldable lens implantation as compared to the other categories. (Table 5d).

#### 3.5 Combined data

Upon combining the data from all three centres (AIIMS Delhi, Shroff Delhi and Shroff Vrindavan), we had six different combinations of cataract surgery with lens implantation (ECCE, SICS and Phaco with either PMMA or foldable lens implantation).

*EQ-5D-5L Results_ Combined*. The overall changes in QALY after the surgery, were positive for all the combinations except for ECCE and SICS surgery with foldable lens implantation (Table 9). One possible reason for such results, could be number of patients undergoing these interventions. For ECCE with foldable lens and SICS with foldable lens, there were only 3 patients each, resulting into a QALY gain of -1.18 and 0.0 respectively. Similarly, number of patients undergoing ECCE with rigid lens implantation and Phaco with rigid lens implantation, were 28 and 33 respectively. Possibility of this data to be different, in case of a higher sample size, cannot be denied.

**Table 9:** EQ-5D results of overall combined data (AIIMS Delhi + Shroff Delhi+ Shroff Vrindavan).

N	Category	Mean									
		Age	Pre Utility	Post Utility	Change	Pre QALY	Post QALY	Change QALY (p-value)	Pre VAS	Post VAS	Change
**3**	**ECCE & Foldable**	59.00	0.76	0.69	-0.07	15.19	14.01	-1.18 (0.88)	**43.33**	**70**	**26.67**
**28**	**ECCE & PMMA**	60.00	0.77	0.90	0.14	14.98	17.49	2.50 0.006	**55.36**	**77.79**	**22.43**
**327**	**Phaco & Foldable**	60.04	0.82	0.93	0.11	16.00	18.25	2.25 <0.01	**59.97**	**85.21**	**25.24**
**33**	**Phaco & PMMA**	61.18	0.85	0.85	0.01	15.30	15.53	0.23 0.75	**50.15**	**76.21**	**26.06**
**3**	**SICS & Foldable**	55.67	0.96	0.96	0.00	22.15	22.15	0.00 0.42	**68.33**	**100**	**31.67**
**123**	**SICS & PMMA**	62.07	0.89	0.98	0.09	15.62	17.30	1.68 <0.01	**57.06**	**92.76**	**35.71**
**31**	**ECCE**	59.90	0.77	0.88	0.12	15.01	17.15	2.15 0.03	**54.19**	**77.03**	**22.84**
**360**	**Phaco**	60.15	0.82	0.92	0.10	15.94	18.00	2.06 <0.01	**59.07**	**84.38**	**25.32**
**126**	**SICS**	61.91	0.89	0.98	0.09	15.77	17.42	1.64 <0.01	**57.33**	**92.94**	**35.61**
**333**	**Foldable**	59.99	0.82	0.93	0.11	16.05	18.25	2.20 <0.01	**59.89**	**85.20**	**25.31**
**184**	**PMMA**	61.59	0.86	0.95	0.08	15.46	17.01	1.55 <0.01	**55.56**	**87.52**	**31.96**
**517**	**Overall**	60.56	0.84	0.94	0.10	15.84	17.81	1.97 <0.01	**58.35**	**86.03**	**27.68**

**Fig 1:**
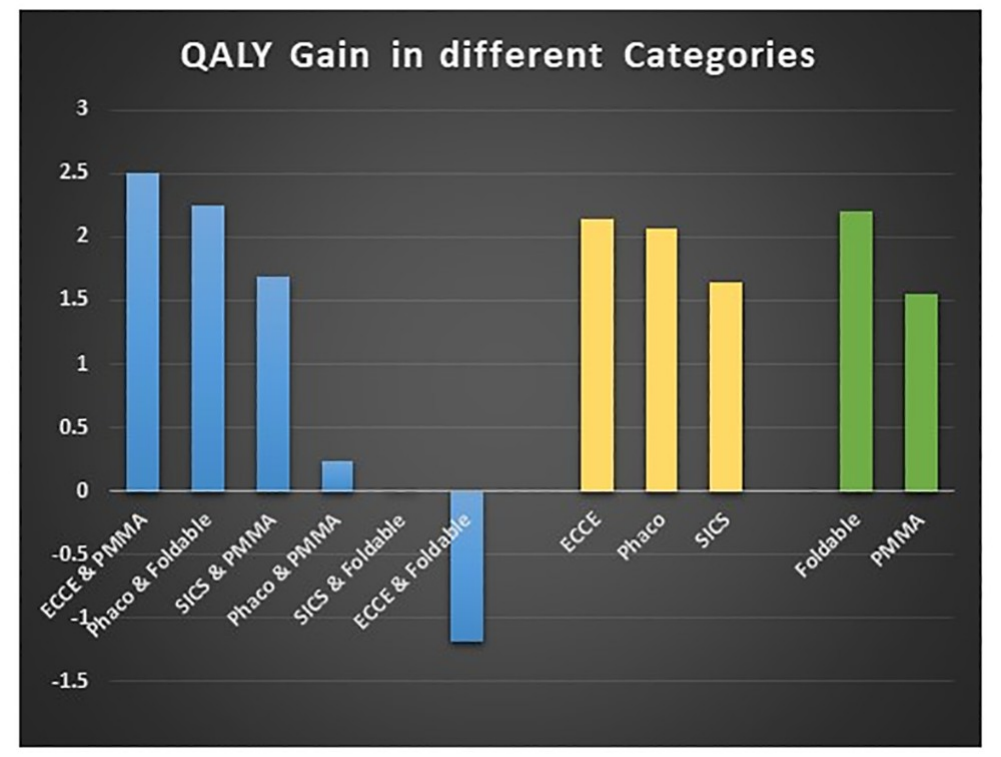
Graph representing the QALY gain in EQ-5D results of overall combined data (AIIMS Delhi + Shroff Delhi+ Shroff Vrindavan).

Due to inadequate sample size, if we exclude all categories except Phaco with foldable lens (n = 327) and SICS with rigid lens (n = 123) for comparison of QALY gain, the combined data from all three centres showed that the QALY gains in patients undergoing phacoemulsification with foldable lens implantation (2.25 QALY) were significantly higher (0.57 QALY) as compared to SICS with PMMA lens implantation (1.68 QALY). (Table 9 and Fig 1).

**Fig 2:**
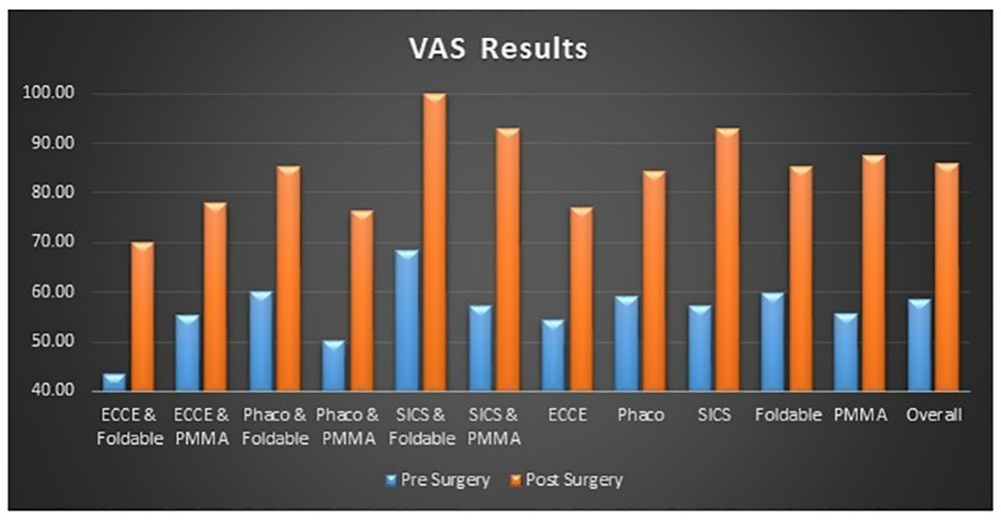
Graph representing pre and post surgery EQ5D visual analogue scale (EQ-5D-VAS) results for overall combined data (AIIMS Delhi + Shroff Delhi+ Shroff Vrindavan).

*EQ-5D VAS Results for combined data*. There is a marked increase in the VAS scores for all the categories with the increase in VAS scores being about 30–40% post-surgery (Tables 4, 6–9 and Fig 2).

**Fig 3:**
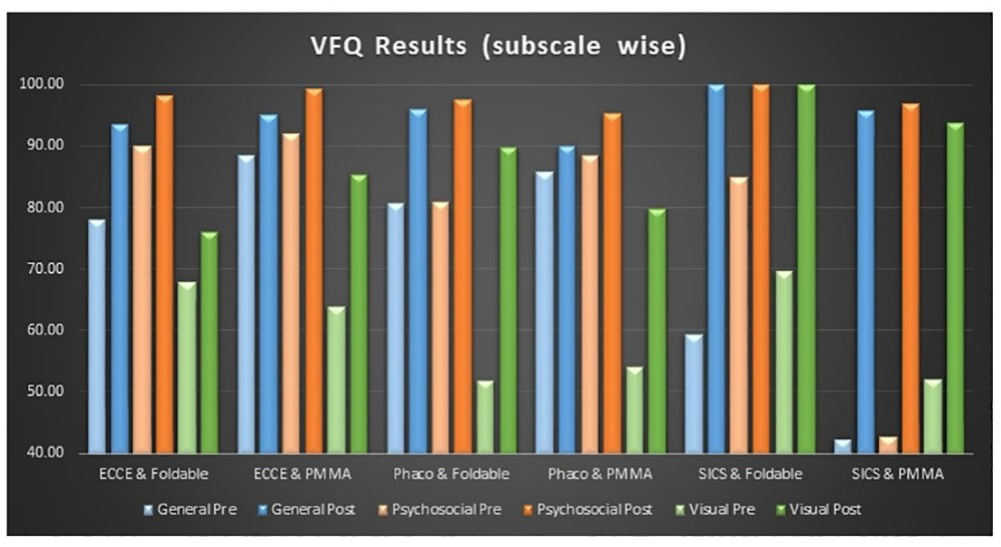
Pre and post surgery scores for IND-VFQ-33 subscales for different combinations of surgery and IOLs for combined data (AIIMS Delhi + Shroff Delhi+ Shroff Vrindavan).

*IND VFQ 33 Results_ Combined data*. Combined results indicated a marked increase in the scores of all three subscales including general functioning, psychosocial impact and visual symptoms of the questionnaire for each type of surgery and lenses. However, there were marked variations between different categories. Highest improvement however, in all three subscales of IND-VFQ-33 tool were clearly observed for SICS with PMMA lens implantation. (Table 5e and Fig 3).

### 4. Visual outcomes

Complete follow up data on visual acuity and EQ-5D tool was available for 511 patients (254 from AIIMS-New Delhi, 125 from Shroff -Delhi and 132 from Shroff -Vrindavan). Follow up UCVA data shows that out of 511 operated eyes 390 (76.32%) have achieved good unaided vision after their surgery, while borderline and poor UCVA were observed in 99 (19.37%) and 22 (4.30%) eyes, respectively. The results show that maximum QALY gain was observed in people where post-operative UCVA were borderline (2.335 QALY), followed by people with good UCVA (1.98 QALY) and least QALY gain observed for people with poor UCVA (0.75 QALY). (Table 10).

**Table 10:** Pre and post surgery uncorrected visual acuities (UCVA) and QALY gain observed for patients undergone different surgeries and lens implantation.

Post-surgery follow-up UCVA n (%)	Pre-surgery UCVA	Surgery (n)	Lens (n)
QALY gain	n (%)		
Good (6/6–6/18)	Good 39 (10%)	Phaco (36)	Rigid (1)
			Foldable (35)
		SICS (3)	Rigid (3)
390 (76.32%)	Borderline 150 (38.46%)	Phaco (126)	Rigid (6)
			Foldable (120)
		SICS (24)	Rigid (22)
			Foldable (2)
QALY gain (1.98)	Poor 201(51.53%)	Phaco (121)	Rigid (5)
			Foldable (116)
		ECCE (9)	Rigid (8)
			Foldable (1)
		SICS (71)	Rigid (70)
			Foldable (1)
Borderline (6/24–6/60)	Good 2 (2.02%)	Phaco (2)	Foldable (2)
99 (19.37%)	Borderline 33 (33.33%)	Phaco (24)	Rigid (5)
			Foldable (19)
		SICS (9)	Rigid (9)
QALY gain (2.335)	Poor 64 (64.64%)	Phaco (36)	Rigid (9)
			Foldable (27)
		ECCE (15)	Rigid (14)
			Foldable (1)
		SICS (13)	Rigid (13)
Poor (<6/60)	Good 1 (4.54%)	Phaco (1)	Foldable (1)
22 (4.30%)	Borderline 3 (13.63%)	Phaco (3)	Rigid (2)
			Foldable (1)
QALY gain (0.75)	Poor 18 (81.81%)	Phaco (7)	Rigid (4)
			Foldable (3)
		ECCE (7)	Rigid (6)
			Foldable (1)
		SICS (4)	Rigid (4)

Out of 511 operated eyes, maximum patients (55.38%) had their pre-operative UCVA as <6/60, followed by patients (36.39%) who had their pre-operative UCVA as 6/24–6/60 and least number of patients (7.82%) had their pre-operative UCVA as 6/6–6/18. In all three post-surgery follow-up UCVA categories (good, borderline and poor), maximum patients had their pre-operative UCVA as <6/60, followed by patients who had their pre-operative UCVA as 6/24–6/60 and least number of patients had their pre-operative UCVA as 6/6–6/18. Least QALY gain (0.75 QALY) was observed in patients with poor outcome after surgery where most patients (81.81%) had their pre-operative UCVA as <6/60 and showed no improvement in visual acuities after the surgery. Maximum QALY gain was observed in patients with borderline outcome after surgery where 98% patients had their pre-operative UCVA as 6/24–6/60 or <6/60. (Table 10).

The study observed that follow up UCVA improvements were better in case of Phaco and SICS as compared to the ECCE surgery. Results also showed that UCVA improvements were not different between phacoemulsification and SICS surgery. In both phacoemulsification and SICS groups more than 79% patients have achieved good unaided vision after the surgery. This highlights the finding of the study that visual outcomes after cataract surgery were independent of the type of surgery (phaco vs. SICS) and types of lens implanted (rigid or foldable). (Table 11).

**Table 11:** Improvement in post-surgery follow up uncorrected visual acuities observed for different surgeries.

	Post-surgery follow-up UCVA			
Surgery	Good (6/6–6/18)	Borderline (6/24–6/60)	Poor (<6/60)	Total
	n (%)	n (%)	n (%)	
**Phaco**	283 (79.49%)	62 (17.42%)	11 (3.09%)	356
**SICS**	98 (79.03%)	22 (17.74%)	4 (3.23%)	124
**ECCE**	9 (29.03%)	15 (48.39%)	7 (22.58%)	31
**Total**	390	99	22	511

#### 4.1 Refractive outcomes

As shown in Table 12, follow up UCVA in 76.32%, 19.37%, and 4.30% of the eyes were VA ≥6/18, 6/18–6/60 and < 6/60, respectively. There were more patients that had poor pre-surgery UCVA (<6/60) in the borderline post-surgery UCVA group (64.64%) than in the good post-surgery UCVA group (51.53%). After the refraction, follow up BCVA showed that 94.32% patients have achieved good outcome, and only 3.32% and 2.34% patients remaining in the borderline and poor outcomes categories respectively. (Table 12).

**Table 12:** Uncorrected (UCVA) and best corrected (BCVA) visual acuities 4 weeks after surgery.

Visual Acuity	UCVA	BCVA
**Good** (6/6–6/18)	390 (76.32%)	482 (94.32%)
**Border Line** (6/24–6/60)	99 (19.37%)	17 (3.32%)
**Poor** (< 6/60)	22 (4.30%)	12 (2.34%)
**Total**	511 (100%)	511 (100%)

## Discussion

Cataract surgeries have continuously been evolving to make the procedure more accurate, convenient and easier to perform with minimal post-surgical complications [31]. Among the many procedures available for cataract surgery, ECCE involves a limbal incision and an anterior capsulotomy, where lens nucleus and cortex are delivered by manual expression [31]. SICS, a variant of ECCE, involves a relatively smaller incision as compared to ECCE [31]. Phacoemulsification involves an anterior opening in the lens capsule with the lens being emulsified by an ultrasonic hand piece and then, aspirated through a 2·2–3·2 mm incision, before an intraocular lens is implanted into the capsular bag [31].

EQ5D is an established tool for measuring generic QoL and being used in countries like UK for decision making, however, the limitations of using the EQ-5D is widely acknowledged for vision related disorders as the instrument lacks a particular domain in measuring vision problems [32–35]. Until a patient is not severally visually impaired, he or she could be quite well in terms of EQ-5D dimensions- mobility, self-care, usual activities, pain/discomfort and anxiety/depression. Therefore, the IND-VFQ-33, which is a much-detailed questionnaire as compared to EQ5D was also administered to patients in this study. With its three subscales, which are a 21-item section for general function, a 5-item section for psychosocial impact and a 7-item section for visual symptoms, it effectively covers the change in the visual aspect of health for the patient [36]. Again, as with most questionnaires for patient reported outcome measures (PROMs), which rely on the patient’s own preferences and judgement, it is still a matter of debate to say which tool is more reliable in the current study scenario.

Regardless of which surgical procedure was followed for cataract removal or what type of lens was implanted thereafter, there was an overall gain of 1.97 QALYs as per the EQ5D questionnaire (n = 517) (Table 9). Similarly, favorable changes with a marked rise in the subscale scores were seen in all the three subscales of IND-VFQ-33 tool for a pool of 519 patients (Table 5). This data reconfirms many previous studies in saying that cataract surgeries are clinically very effective in restoring the vision as well as in improving patient’s quality of life [37–42].

All three types of surgical procedures (ECCE, Phaco and SICS) analyzed in the study have shown to make a marked improvement in generic (QALY gain shown by EQ-5D results) as well as vision related quality of life (gain in individual subscales in IND-VFQ-33 tool). Present study shows that QALY gains in patients undergoing phacoemulsification with foldable lens implantation (2.25 QALY) were significantly higher (0.57 QALY) as compared to SICS with PMMA lens implantation (1.68 QALY). Literature available on HRQoL studies comparing phacoemulsification with SICS surgeries is scarce. The only study comparing phacoemulsification with SICS surgery was conducted as a cost effectiveness analysis in India [42]. This was a prospective randomized controlled trial performed in a tertiary care hospital setting. Preoperative and postoperative LogMAR visual acuity (VA), visual function-14 score and their quality-adjusted life years were obtained. QALYs and VFQ Scores for both the SICS with rigid lens and Phaco with foldable lens groups achieved comparable outcomes in terms of change in LogMAR VA, VF-14 score and QALYs. However, the study was performed in a small sample size of 52 patients only, who were randomly assigned to Phaco and SICS surgeries. The study does not describe how QALY values were assigned in different groups [42].

Though there is dearth on literature available on comparative studies, there are a few studies that provide utility values on pre and post phacoemulsification cataract surgery using EQ-5D as an instrument [38, 43, 44]. We compared results from these studies to the phacoemulsification results observed in the present study. All three published studies reported a QoL gain of 0.4–0.5 after phacoemulsification surgery (Table 13). In present study we observed a higher gain in QoL (0.10) as compared to these studies. There could be many reasons for this variation. A much higher sample size in our study could also be a reason. Besides, in the present study we have used EQ-5D-5L, whereas other studies have used EQ-5D-3L as an instrument. This means, The EQ-5D instrument used in this study has five levels for each dimension, whereas in other studies there were only three levels for each dimension. EQ-5D-5L is known to have superior psychometric properties being more sensitive to patient’s responses as it reduces the ceiling effect and has higher discriminatory power in patients with chronic diseases [45, 46]. Therefore, EQ-5D-5L might possibly be more accurate in capturing the HRQOL benefits of cataract surgery.

**Table 13:** QoL gains after phacoemulsification surgery observed in different studies.

	Country	Sample size	Tool used	Pre surgery QoL	Post-surgery QoL	Gain in QoL
Griffith et al. [32]	Zambia	77	EQ-5D 3L	0.782	0.832	0.05
				SD 0.150	SD 0.129	
Hiratsuka et al. [37]	Japan	138	EQ-5D 3L	0.84 ± 0.15	0.89 ± 0.15	0.05 ± 0.15
Le et al. [38]	India	292	EQ-5D 3L	0.84	0.88	0.04
Present Study	India	360	EQ-5D 5L	0.82	0.92	0.10

Both type of lens (rigid PMMA and foldable lens) analyzed in this study, have shown to make a marked improvement in generic as well as vision related quality of life. However, the generic quality of life was found to be better in case of foldable lens as compared to the rigid lens. As far as the vision related quality of life associated with lens implanted is concerned, the study shows visual symptoms scores were highest in case of foldable lens while the other two subscales-general functioning and psychosocial impact were best improved by rigid lens. A randomized controlled trial conducted at Sagarmatha Choudhary Eye Hospital, Lahan, Nepal, compared the outcomes of phacoemulsification with either a 2.5-mm clear corneal incision and a foldable intraocular lens or a 5-mm sclera-corneal tunnel incision and a rigid PMMA lens [47]. Cost of the foldable IOL was found many times higher than the PMMA IOL with no additional clinical benefit when implanted after phacoemulsification [47]. We did not find any study on HRQoL that directly compares foldable lenses to the rigid PMMA lenses providing utility scores.

Though there is abundant literature available on clinical effectiveness of cataract surgery, only a few studies were found reporting quantitative QALY data [37–39, 48]. Most of the studies provided HRQoL results in terms of improvement in performing visual activities, daily routine activities, social wellbeing etc but utility scores are not mentioned [49–54]. There are some studies where QoL results are given for different dimensions, e.g. it is given on mobility, self-care, usual activities, pain/discomfort and anxiety/depression if EQ5D is used as an instrument but utility weights were not assigned against the overall health state and therefore QALYs were also not estimated [55, 56].

As far as visual acuity outcomes after the surgery are concerned, this study reconfirms previous studies in saying that both SICS and Phaco stands out to be better as compared to ECCE [57, 58]. Another key finding from the present study is that similar improvements in uncorrected visual acuities are observed in both the phacoemulsification and in SICS surgery group. There are ample evidences suggesting that SICS and Phaco results in almost similar outcomes in terms of post-operative visual acuity (both UCVA and BCVA) and post-operative complications (astigmatism, endothelial cell loss, post-operative capsular rupture, and corneal edema) [59–62].

It is understandable that the least QALY gain was observed in patients with poor outcome with no improvement in visual acuities after surgery. However, patients with post-operative borderline UCVA had higher QALY gain than the group with the good post-operative UCVA. It was observed that post-operative borderline UCVA group had 98% patients with pre-operative UCVA as 6/24–6/60 or <6/60. (Table 10). Our study highlights that patients appreciate gain in number of Snellen’s visual acuity lines more than final unaided visual acuity.

To the best of our knowledge, this is the first comprehensive study that provides HSUVs for different surgical procedures, lenses and for the combination of surgery with lens implantation for cataract procedures for such a large sample size. The study provides both generic as well as condition specific (vision related in this case) HRQoL results using established patient reported tools. The study also provides data on visual outcomes after the surgery in terms of uncorrected and best corrected visual acuities after the surgery along with associated QALY gains. Though all three study centers were in north India, patient population represented quite a good mix of patients from different regions and background from all over the country, as these centers caters to huge load of patient every day.

The present study has several limitations also. The follow-up for the questionnaires was only about 64% due to some patients not answering some questions or entirely skipping the questionnaire in the follow-up phase. In these cases, we had to exclude the questionnaires from analysis (both pre- and post-surgery). Sample size in different categories varied a lot as different institutes have different standards of practice and the bulk population is treated with phacoemulsification nowadays. Our biggest limitation for this study was country specific value sets, that is a major feature of the EQ-5D instrument, facilitating the calculation of QALYs were not available for India, that led us using EuroQoL certified Indonesian value set, considering it the next best available resource for the purpose. Though Indonesian population is quite similar to India, it can still have significant impact on estimation on QoL scores. Another limitation of the study is not being able to analyze the data in terms of socio-economic status of the patients, which could have a significant impact on patient’s judgement itself for responding to any patient reported outcome tool. These questionnaires rely on the patient’s responses and thus are very subjective, based on the patients’ perception of their own health status [63–65]. A patient from a lower socioeconomic stratum might have a higher pain tolerance than those from the higher ones based on their day-to-day activities and their coping mechanisms [63–65].

Considering the data from the present study, any of these surgical procedure and lens combination may help the healthcare system in the management of cataract patients and restoring their vision. However, for practical reasons, it seems more appropriate to use foldable lens with phaco as the incision size is smaller and rigid lens with ECCE and SICS due the larger incision size. Though its conventional to compare the surgeries and lenses in terms of costs and health outcomes for an economic evaluation study done for the purpose of deciding resource allocation, there could be many aspects other than cost effectiveness worth considering with equal emphasis while making an evidence informed decision. Availability of infrastructure, expertise of surgeons practicing in that area, accessibility of the services for remotely located patients are a few points to be kept in mind while making a careful decision at the local level.

This study provides health state utility values for cataract procedures, lenses and combinations of surgery with lens, along with data on vision related quality of life that could be a highly useful resource for future economic evaluation studies. Information presented here could be used by a wide group of users including researchers, public health experts, ophthalmologists, policy makers, insurance provider, etc.
